# Improvement of Ocular Surface Disease by Lateral Tarsoconjunctival Flap in Thyroid-Associated Orbitopathy Patients with Lid Retraction

**DOI:** 10.3390/jpm12050802

**Published:** 2022-05-16

**Authors:** Chih-Kang Hsu, Meng-Wei Hsieh, Hsu-Chieh Chang, Yi-Hao Chen, Ke-Hung Chien

**Affiliations:** 1 Department of Ophthalmology, Tri-Service General Hospital and National Defense Medical Center, Taipei 114, Taiwan; chikanghsu@gmail.com (C.-K.H.); doc30879@mail.ndmctsgh.edu.tw (Y.-H.C.); 2 Department of Ophthalmology, Tri-Service General Hospital Songshan Branch, Songshan, Taipei 105, Taiwan; 3 Department of Ophthalmology, Taoyuan Armed Forces General Hospital, Taoyuan 325, Taiwan; nsinkoph0311@gmail.com; 4 Department of Nursing, Tri-Service General Hospital, National Defense Medical Center, Taipei 114, Taiwan; n3197001@gmail.com; 5 Graduate Institute of Nursing, College of Nursing, Taipei Medical University, Taipei 110, Taiwan

**Keywords:** thyroid-associated orbitopathy, hyperthyroidism, lid retraction, dry eye, ocular surface disease

## Abstract

There is a high incidence of ocular surface disease (OSD) in thyroid-associated orbitopathy (TAO) patients as a result of incomplete eyelid closure and chronic inflammatory eyelid status. This study was performed to evaluate the efficacy of a lateral tarsoconjunctival flap (LTF) in improving OSD in TAO patients from the perspective of correcting eyelid closures. As a study design, TAO patients were enrolled in this study to evaluate OSD perioperatively before they were scheduled for LTF surgery. Additional lid surgery was also recorded. The outcome was evaluated with perioperative OSD measurements and tear inflammatory mediators at baseline and one month and three months postoperatively. As a result, 42 patients (5 male, 37 female) underwent LTF surgery, and 13 patients received medial pretarsal support with collagen grafts. Eleven patients underwent blepharotomy, and 6 patients received Botox injections for upper lid retraction. The mean age of the participants was 46.4 years old, and the mean follow-up period was 10.6 months. Their clinical activity score (CAS) at the time of the operation was 2.1. Dry eye parameters, such as ocular surface disease index, tear breakup time, Schirmer’s I test score, and meibomian gland dropout were all significantly improved 3 months postoperatively. Tear osmolarity and inflammatory mediators in tear fluid, such as IL-6, IL-8, IL-18 and MCP-1, were also significantly improved after the procedures. After the surgery, all complications were mild and temporary. As a conclusion, LTF could provide TAO patients with both lid retraction correction and improvement of ocular surface disorders. Dry eye parameters significantly improved 3 months postoperatively. This method can serve as an alternative treatment option for lid correction in TAO patients.

## 1. Introduction

Thyroid-associated ophthalmopathy (TAO) is a complicated disorder resulting from autoimmunity of the orbital tissues with characteristics of chronic inflammation of retrobulbar tissues, adipogenesis, and the accumulation of glycosaminoglycans in the extraocular muscles (Salvi & Campi, 2015) [1]. Clinical manifestations of TAO vary based on disease severity and the involved tissues. Common presentations include dry eye, lid retraction, corneal erosion, diplopia, visual impairment, and even secondary glaucoma (Weetman, 1991) [2]. Medication to control underlying thyroid function is the mainstay of the treatment, but up to 20% of TAO patients eventually receive surgical intervention, including orbital decompression, strabismus correction and lid retraction correction (Baldeschi et al., 2006) [3]. In the past decade, the IGF-1 receptor inhibitor teprotumumab (Tepezza) was approved to treat moderate-to-severe active TAO and changed the management protocol for TAO patients (Neag & Smith, 2021) [4].

Dry eye is one of the most common symptoms in TAO patients (Sun et al., 2021) [5]. A total of 65–85% of TAO patients are found to have complaints of dry eye symptoms (Selter et al., 2015) [6]. In addition to an increase in the interpalpebral fissure and accelerated tear film evaporation (Moura Brasil et al., 2005) [7], there are more complicated pathologies found in TAO that cause dry eye. Corneal sensitivity is found to decrease even without apparent proptosis in early TAO (Achtsidis et al., 2013) [8]. In addition, to tear film abnormalities found in TAO, meibomian gland dysfunction (MGD) is highly prevalent in TAO patients, affecting 68% of people with this disease (Tirakunwichcha et al., 2016) [9]. In a recent study, after classical management of orbital decompression, MGD scores, rather than other dry eye parameters, were noted to be reduced significantly (Takahashi et al., 2021) [10].

Lid retraction is a common finding in TAO patients with the displacement of the lid from the normal position, overriding the limbus with scleral show present (JR et al., 2020) [11]. Usually, upper lid retraction is managed by either medication injection or blepharotomy, (Karabulut et al., 2020; Kazim et al., n.d.; Salour et al., 2010) [12,13,14] and there are various choices for treating low lid retraction. Although the cause of lower lid retraction in TAO is suggested to be proptosis and not lamellar shortening (Rajabi et al., 2013) [15], several surgical techniques are effective in correcting lower lid retraction on lid lamellae (Kersten et al., 1990a; Lang & Wei, 2013; Mourits & Koornneef, 1991; Olver et al., 1998b) [16,17,18,19]. The lateral tarsoconjunctival flap (LTF) is a modification of lateral tarsorrhaphy that links a partial tarsal graft over the upper lid with the posterior lamina over the lower lid margin at the distal end of the lids. In this study, we evaluated changes in ocular surface status after lid retraction correction with the LTF procedure. Parameters related to ocular surface status were compared perioperatively.

## 2. Materials and Methods

Patients with thyroid-associated orbitopathy (TAO) were invited to enter the study when they consulted for lid surgery to correct their lagophthalmopathy from January 2020 to December 2021. The study was approved by the Institutional Review Board of Tri-Service General Hospital (C202105036). Written informed consent was obtained from all subjects at the time of enrollment.

Inclusion criteria of the study were (1) age at least 20 years old; (2) patients with TAO presented with lower lid retraction and completed prior steps of management including orbital decompression and strabismus correction (3) patients with hyperthyroidism had stable disease with euthyroid status achieved at least 6 months before their lid surgery. The exclusion criteria were the retraction of the lower lid from causes other than TAO. Data collected included age, sex, clinical activity scores of TAO, and history. Preoperative and postoperative evaluations included visual acuity (VA), intraocular pressure (IOP), slit lamp exam, LipiView (Johnson & Johnson, New Brunswick, NJ, USA) records and ocular surface disease evaluations, including ocular surface disease index (OSDI) questionnaire, ocular surface staining including the cornea and conjunctival staining with rose bengal staining system, [20] tear break up time (TBUT) (a value of 10–35 s was considered normal), Schirmer test and gland drop out (adopted the Meibograde grading system) [21]. Additionally, tear osmolarity and tear fluid inflammatory mediators, such as IL-6, IL-8, IL-18 and MCP-1, were measured perioperatively. In patients who underwent surgeries in both eyes, we recorded the information of the right eye for statistics; moreover, healthy volunteers were enrolled for providing values of tear osmolarity and tear fluid inflammatory mediators as a control group.

The indications for LTF surgery were as follows: (1) diagnosis of thyroid-associated orbitopathy; (2) balanced exophthalmometry status with an interocular difference of less than 2 mm and less than 20 mm in both eyes; and (3) inactive disease with a euthyroid state. Surgery was performed after a euthyroid state, medication control, and stable ophthalmic signs with regular follow-up for more than 6 months were achieved. Grading of lower lid retraction was determined by the inferior scleral show in the primary position. 0–3 mm scleral show was at mild grade and could be corrected with LTF alone. Scleral show more than 3 mm was at a severe grade and a collagen graft was applied together with the LTF procedure.

**Figure 1:**
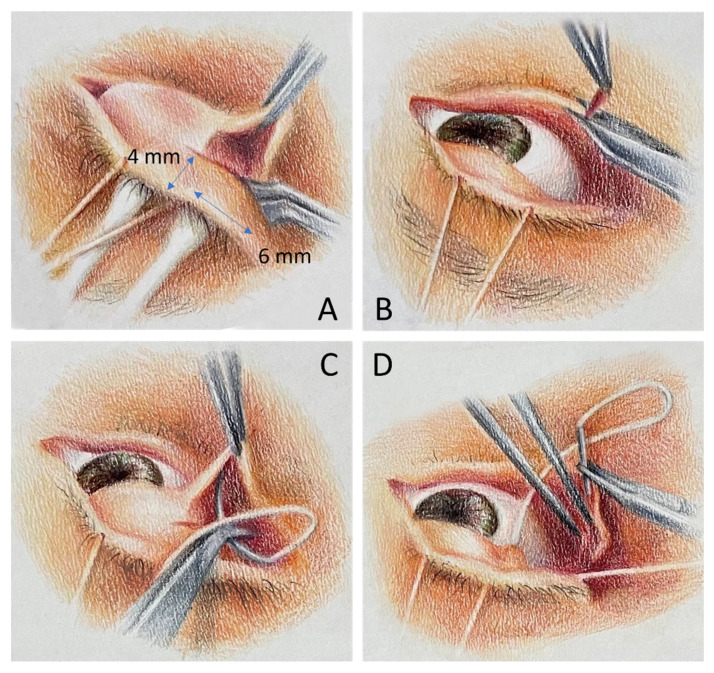
Surgical procedures illustration of lateral tarsoconjunctival flap (LTF). Briefly, an area measuring 4 mm (vertical length) × 4–6 mm (horizontal length) from the lid margin was marked over the tarsal conjunctiva to create a partial tarsal graft after the upper lid was everted (**A**). Then, the lateral 4–6 mm aspect of the lower lid margin was denuded posterior to the grey line with 15-blade or Westcott scissors (**B**). Finally, one or two interrupted, 5-0 Vicryl sutures were placed at the inferior edge of the upper eyelid flap and the denuded lower eyelid margin to create the LTF (**C**,**D**).

All surgeries were performed under local anaesthesia by one surgeon (Ke-Hung Chien, MD). The surgical procedures were adopted as described by Conger et al., with modifications. (JR et al., 2020) [11] Briefly, an area measuring 4 mm (vertical length) × 4–6 mm (horizontal length) from the lid margin was marked over the tarsal conjunctiva to create a partial tarsal graft after the upper lid was everted (Figure 1A); then, the lateral 4–6 mm aspect of the lower lid margin was denuded posterior to the grey line with 15-blade or Westcott scissors (Figure 1B).

One or two interrupted, 5-0 Vicryl sutures were placed at the inferior edge of the upper eyelid flap and the denuded lower eyelid margin (Figure 1C,D).

Additional procedures were permitted in the current study to ensure a better lid position if the patient was found with horizontal laxity or medial lower lid retraction at the time of the surgery:

(1) To enforce horizontal force, we performed the tarsal strip technique in some patients who presented with horizontal laxity at the time of surgery.
(2) To improve medial support of the lower lid, we added a collagen graft at the pretarsal plane in patients who presented with residual medial lower lid retraction despite LTF during the surgery or inferior scleral show more than 3 mm in the preoperative evaluation.

To measure the inflammatory mediators in the tears, patients’ tears were collected with the following protocol. Kimwipes pre-cut in 1.0*1.0 cm were used to absorb tears in the lower conjunctival fornix after dripping 20 µL normal saline by a pipette; then, the kimwipes were soaked in PBS in a microtube and the sample was collected after the microtube which was put in a 1.5 mL Eppendorf and underwent centrifugation (2000× *g*, 4 °C, 15 min). The sample was sent for Milliplex Human Cytokine/Chemokine/Growth Factor Panel (Merck Millipore, Billerica, MA, USA) for measurement of IL-6, IL-8, IL-18 and MCP-1 in our patients. And the tear osmolarity was measured by the I-Pen (I-Med Pharma, Saint-Laurent, QC, Canada) with a chip which was attached to the main device contacting the tears in the conjunctival sac of the patients. A series of tests were performed following the sequences below and had done each of the tests at 15 min apart, collecting tears for tear osmolarity and inflammatory mediators as the first step, then imaging tests such as LipiView and Gland drop-out evaluation, and fluorescence-using tests like ocular surface staining test, tear break up time and Schirmer test in the last step to avoid test interference.

Follow-up visits were arranged at one week, one month, and three months postoperatively. Clinical outcome measurements and adverse effects were recorded. The efficacy of LTF surgery in improving OSD in these patients was analyzed using SPSS software version 18.0 for Windows (SPSS Inc. Released 2009. PASW Statistics for Windows, Version 18.0. Chicago: SPSS Inc.). Wilcoxon signed-rank test was applied to test the difference among parameters at different time points. All data are presented as the mean ± standard deviation. Values of *p* < 0.05 were considered statistically significant.

## 3. Results

A total of 42 TAO subjects who underwent LTF surgery at Tri-Service General Hospital were included in the study. The 42 subjects comprised 37 females and 5 males, with a mean (SD) age of 48.37 (12.3) years and an age range of 30.2 to 57.6 years. There were 39 patients (92.9%) who developed TAO because of underlying hyperthyroidism, with 2 cases of Hashimoto’s thyroiditis and 1 case of consecutive hypothyroidism (from thyroidectomy). The mean (SD) follow-up time of the patients was 10.6 (4.2) months, ranging from 6.1 months to 20.4 months (Table 1). All patients completed preoperative and one-month and three-month postoperative visits.

Regarding the patients’ background prior to the surgery, 24 patients (57.1%) had a history of smoking. All patients had been receiving medical treatment and attained a euthyroid state at least 6 months before surgery; 26 patients (61.9%) had a history of prior usage of corticosteroids, including via oral and intravenous routes; 5 patients (11.9%) underwent radioactive iodine treatment; 1 patient (2.4%) underwent radiotherapy. The mean duration of TAO before LTF surgery was 31.4 months. There were 33 patients (78.6%) who had prior ocular surgery experience, with 29 who had surgeries related to thyroid ophthalmopathy, including decompression and strabismus corrections; 5 who had lid bag surgeries; and 1 who had surgery to remove cataracts. The mean clinical activity score was 2.1 (SD 0.6) (Table 1). In the additional procedures performed with LTF surgery, there were 13 patients who received medial pretarsal support with collagen grafts, 11 patients who underwent upper blepharotomy, and 6 patients who received Botox injection for upper lid retraction.

**Figure 2:**
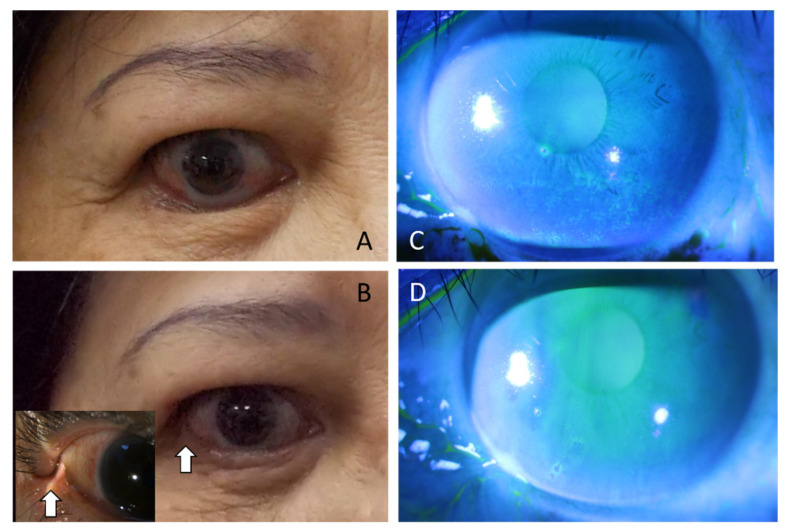
External ocular photos showed lower lid retraction with 2–3 mm scleral show and entropion (**A**). Three months postoperatively, entropion with retraction was corrected with LTF which was barely seen in the lateral canthus (pointed by arrowhead) (**B**). Diffuse superficial punctate kearatopathy was seen over middle to inferior third of the cornea surface before LTF surgery (**C**). Three months postoperatively, the cornea surface was recovered without apparent corneal damage noted (**D**) (Different patients between (**A**,**B**) and (**C**,**D**)).

**Figure 3:**
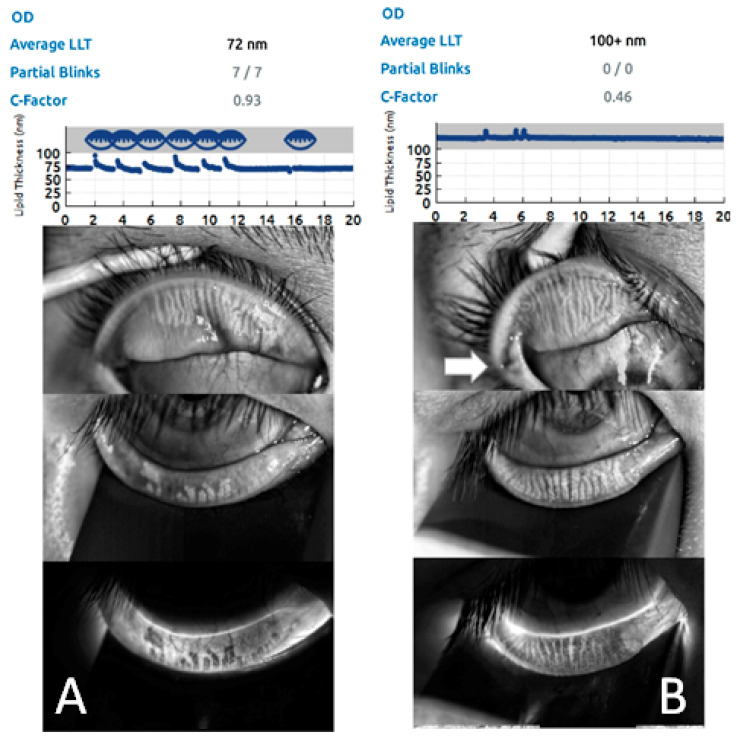
Blinking efficiency improved after the LTF procedure. Before the procedure, there were significantly Meibomian gland drop-outs both in the upper and lower lids with several partial blinks (incomplete blinking) (**A**). Three months after the procedure (the LTF was marked by the arrow), blinking efficiency was significantly improved with fewer meibomian gland drop-outs and no incomplete blinking (**B**).

To evaluate ocular surface status changes after LTF, we performed a series of examinations at time points before the operation (baseline) and 1 month and 3 months post-operation. Regarding the results of the subjective measure OSDI, the mean value was 48.7 (SD 10.8) preoperatively, 36.48 (SD 9.80) at one month after the operation, and 24.32 (SD 8.24) at 3 months postoperatively. Additionally, objective measurements, such as the TBUT and Schirmer’s I test, revealed significant improvement in patients after 3 months. In TBUT, there was a mean break-up time of 3.9 s (SD 0.6) at baseline, which improved at 4.6 s (SD 0.9) one month postoperatively and significantly improved to 6.8 s (SD 1.3) at the 3-month postoperative visit (Table 2); additionally, we performed fluorescence staining to evaluate OSD status perioperatively. Corneal and conjunctival staining all improved from 4.6 (corneal staining scores) and 5.2 (conjunctival staining scores) at the baseline, to a significant difference of 3.7 and 3.0 at 3 months postoperatively, respectively (Figure 2). In addition, we evaluated meibomian glands with gland dropout scores. There was a mean of 4.8 (SD 0.5) at baseline, which improved to 4.1 (SD 0.3) at one month postoperatively and 3.4 (SD 0.5) at three months postoperatively (Table 2) (Figure 3).

TAO patients were found to have elevated inflammatory cytokines in tear and tear osmolarity compared to controls (Iskeleli et al., 2008; Kishazi et al., 2018; Ujhelyi et al., 2012) [20,21,22]. We checked tear osmolarity and the tear inflammatory cytokines IL-6, IL-8, IL-18 and MCP-1 at different time points to determine the changes in these parameters related to the procedure in the timeline. As a result, all parameters were significantly different from the control at baseline (preoperatively). One month after the operation, the parameters were all significantly reduced, and the trend continued to decrease at the 3-month postoperative check-up point. (Table 3)

There were some complications in this cohort. Granuloma over LTF was noted in 2 patients (4.8%), and these patients were successfully treated with topical corticosteroid ointments. One patient (2.4%) had a dehiscence of the LTF graft at the 3-month visit without any discomfort or lid position abnormity. No cases of wound infection were noted in our cohort.

## 4. Discussion

Thyroid-associated ophthalmopathy (TAO) is now viewed as an autoimmunity disorder that causes a series of periocular changes. Among them, lid retraction is difficult to fully correct. Upper eyelid retraction (Dalrymple’s sign) is the most significant periocular appearance of TAO and is listed as one of the major criteria for disease diagnosis (Bartley & Gorman, 1995; Cruz et al., 2013) [23,24]. Lower lid retraction, which is also a common feature of TAO, (Graves’ Eye Disease: Orbital Compliance and Other Physical Measurements-PubMed, n.d.) [25], has drawn less attention, and there is combat on the relation with the pathology of TAO (Day, 1960) [26]; however, lid retractions or malposition is a serious concern for ocular surface care, and may result in overocular exposure and thereby cause increased tear evaporation. More severe complications, such as exposure to keratitis, corneal ulcer, permanent corneal haze and vision impairment, may occur without prompt treatment (Kersten et al., 1990b; Olver et al., 1998a) [27,28]. In this study, we demonstrated ocular surface disease improvement in TAO patients with lid retraction by the transconjunctival flap procedure, which mainly worked by improving ocular blink efficiency and reducing ocular exposure.

The quality of tears may change secondary to lid retraction in TAO patients, and changes in tears are vital in the occurrence of OSD in TAO patients. Generally, the tear breakup time (TBUT) and Schirmer’s I test (SIt) are the most widely applied basic indexes to estimate the condition of tears in assessing tear film stability (TBUT) and the amount of basic secretion of tears (SIt) (Wei et al., 2015) [17]. Wei et al. proposed that the BUT and SIt in the GO group both revealed significant differences compared with the control group (Wei et al., 2015) [17]; their reports implied that the change in tears in TAO patients involved both quality and quantity, which is supported by other reports (Huang et al., 2012; Y. S. Kim et al., 2015; Wu et al., 2016) [18,29,30]. Our report supported the finding that tear quality and quantity were all influenced by TAO status, with the result that there were significantly lower TBUT and SIt values at baseline in our cohort (Table 2). After the tarsoconjunctival flap operation, there were significant improvements in both Sit 1-month and TBUT 3-months postoperatively.

Many studies have found that ocular surface tissues and lacrimal glands are both affected by autoantibodies in TAO (Eckstein et al., 2004; Gupta et al., 2009) [31,32]. Eckstein et al. proposed that the lacrimal gland expresses the receptors of thyroid-stimulating hormones (TSH-R), which is recognized as a target of autoantibodies and results in lacrimal gland dysfunction and reduced tear production; the lacrimal glands are a source of secreted inflammatory cytokines in tears and lead to ocular surface damage (Huang et al., 2012) [30]. Previous studies have shown that some inflammatory cytokines in tears, such as IL-1β, IL-6, IL-8, and IL-13, are elevated in TAO patients compared with healthy patients (Kishazi et al., 2018; Ujhelyi et al., 2012) [20,21]; these inflammatory cytokines are released through external stimuli and then activate MAPK intracellular signalling pathways, resulting in apoptosis, goblet cell loss, and mucin disturbance on the ocular surface to deteriorate dry eye status (Chen, 2019; Ujhelyi et al., 2012) [33,34]. Previous studies have found that the osmolarity of the tear film is also higher in TAO patients (Iskeleli et al., 2008) [35]; the pathology is believed to be from accelerating tear evaporation secondary to proptosis and increasing the width of the palpebral fissure in TAO patients. We applied a lateral tarsoconjunctival onlay flap in this study to correct lower eyelid retraction; the flap created by the proposed procedure connects the upper fornix and the lower lid margin and transmits the supraduction force from the upper fornix into an upward force to raise the lower lid. The results supported previous findings that there were elevated inflammatory markers, such as IL-6, IL-8, IL-18 and MCP-1, at baseline (Table 3). Notably, at the postoperative follow-up visits, these markers were all significantly reduced at the 1-month and 3-month time points postoperatively. Our surgical procedure reduced the palpebral fissure and then progressively lowered the osmolarity and cytokine concentration of the tear. Aqueous and mucous layers affected by evaporation also improved after surgery.

Efficient blinking plays an important role in tear secretion by contraction of the muscle of Riolan, which is driven by blinking and then encourages meibomian gland secretions (Foulks & Bron, 2003; McMonnies, 2011) [36,37]. Incomplete blinking may result in meibomian stasis, obstruction and intraductal inflammation (Knop et al., 2011) [38]. Obstructed meibomian glands developed gland dysfunction and may be accompanied by gland dropout over time. Chronic gland dysfunction secondary to inefficient blinking is a common feature noted in Cranial Nerve VII palsy and TAO patients (Gupta et al., 2009; Takahashi & Kakizaki, 2015) [32,39]. Our surgical procedure corrected the stasis of the meibomian glands by creating more complete and efficient blinking; the benefit of efficient blinking was demonstrated in our prior study with the dynamic upward motion of the lower lid margin during blinking (Chien et al., 2016) [40], and moreover, the upward lower lid helps protect the lower half of the cornea and tear status when blinking. As a result, staining in both the cornea and conjunctiva and meibomian gland dropout, which were caused by incomplete blinking, were all significantly improved after surgery (Table 2). As the OSD improved after the procedure over time, gland dropout status also greatly improved at 3 months after the procedure (Figure 3).

Lower lid retraction in TAO patients may involve the whole lower lid more than just the lateral end. Traditionally, collagen grafts or autologous grafts are used to correct lower lid retraction (Chang et al., 2011; Dailey et al., 2015) [41,42]; however, efficient blinking is not fully restored because there is a lack of upward movement in the lower lid during blinking. In this study, we applied LTF in lower eyelid retraction of TAO as the main procedure and added a tarsal strip procedure in residual horizontal loosening cases and a collagen graft in residual medial lid retraction cases. In our practice in elderly patients, the tarsal strip procedure was performed in cases of mild lower eyelid retraction, while spacer grafts were used in cases of severe lower eyelid retraction (K. H. Kim et al., 2017) [43]. Compared with either the traditional tarsal strip procedure or collagen grafting alone, the current technique provides both horizontal support and a vertical force to elevate the lower lid during blinking (Tao et al., 2014) [44]; in addition, the small flap is well hidden in the lateral canthus to restore a natural appearance. The LTF procedure is an easy procedure that provides both cosmetic and functional benefits.

The main limitation was that there was no control or comparative group of other surgical procedures in this noncontrolled study. Due to the study design as an interventional study, it is difficult to assign patients to a control group; instead, we checked baseline preoperative parameters as a comparison to disclose the efficacy of the procedure in OSD status. Besides, ocular surface disease evaluation results could be interfered with by the sequences of tests done. To reduce the interference, we did each of the tests 15 min apart. To understand the differences more clearly between LTF and other procedures, a more well-controlled randomized control study is needed.

## 5. Conclusions

In TAO patients, OSD may be more difficult to address because of increasing the evaporation of tears from proptosis and eyelid retraction, causing extraocular exposure. Our study revealed a decrease in the concentration of inflammatory mediators in tear fluid and improvement in meibomian gland dropout after the LTF procedure; this procedure provides good cosmesis and preserves the lower cornea by the upward motion of the lower lid while blinking, and the quality and quantity of tears also changed. The LTF procedure is an easy and efficient procedure to help OSD control in TAO patients with lid retraction.

## Figures and Tables

**Table 1:** Demographic characteristics of the patients in the study.

No. of Subjects (N) (%)	42 (100%)
Male (N) (%)	5 (11.9%)
Female (N) (%)	37 (88.1%)
Age at operation (years) (mean) (SD)	46.4 (12.3)
Age at operation (years) (min) (max)	(30.2) (57.6)
Underlying hyperthyroidism (N) (%)	39 (92.9%)
History of prior ocular surgery	33 (78.6%)
Clinical Activity Score at operation (mean) (SD)	2.1 (0.6)
Follow-up period (month) (mean) (SD)	10.6 (4.2)
Follow-up period (month)(min) (max)	(6.1) (20.4)

The *p*-Value in the table is from values compared between Group 1 and Group 2. N = case number. SD = standard deviation.

**Table 2:** Comparison of Parameters on Ocular Surface Disease in Different Time Points.

	Baseline	1 Month	3 Months	*p*-Value
OSDI (mean)(SD)	48.7 (10.8)	36.5 (9.8)	24.3 (8.2)	<0.01 *
TBUT (sec)(mean)(SD)	3.9 (0.6)	4.6 (0.9)	6.8 (1.3)	<0.01 *
Schirmer’s I test (mm)(mean)(SD)	5.4 (3.4)	11.3 (3.7)	14.3 (3.9)	<0.01 *
Corneal staining score (mean)(SD)	4.6 (2.5)	4.0 (2.6)	3.7 (2.1)	<0.01 *
Conjunctival staining score (mean)(SD)	5.2 (1.6)	4.7 (2.3)	3.0 (2.8)	<0.01 *
Gland dropout (mean)(SD)	4.8 (0.5)	4.1 (0.3)	3.4 (0.5)	<0.01 *

The *p*-Value in the table is from values compared between baseline and 3 months postoperatively. SD = standard deviation. OSDI = Ocular Surface Disease Index. TBUT = tear break up time. *p* < 0.05 = significant (*).

**Table 3:** Comparison of Tear Fluid Inflammatory Mediators in Patients Perioperatively.

	Control	Baseline	1 Month	3 Months
IL-6 (mean)(SD)(pg/mL)	1.2 (0.3)	39.6 (9.63)	6.3 (2.1)	1.9 (0.3)
IL-8 (mean)(SD) (pg/mL)	8.0 (2.2)	344.9 (57.2)	59.4 (12.7)	25.6 (8.3)
IL-18 (mean)(SD) (pg/mL)	0.7 (0.1)	8.3 (2.3)	3.0 (0.3)	2.2 (0.4)
MCP-1 (mean)(SD) (pg/mL)	100.1 (13.8)	339.3 (68.8)	279.3 (53.5)	230.4 (48.7)
Tear osmolarity (SD) (mOsm/L)	306.2 (16.4)	321.4 (19.6)	316.2 (14.3)	308.6 (18.5)

SD = standard deviation; IL = interleukin; MCP-1 = monocyte chemoattractant protein 1.

## Data Availability

The datasets used and analyzed during the current study are available from the corresponding author on reasonable request.
